# On-board communication-based relative localization for collision avoidance in Micro Air Vehicle teams

**DOI:** 10.1007/s10514-018-9760-3

**Published:** 2018-05-02

**Authors:** Mario Coppola, Kimberly N. McGuire, Kirk Y. W. Scheper, Guido C. H. E. de Croon

**Affiliations:** 1 Department of Control and Simulation (Micro Air Vehicle Laboratory), Faculty of Aerospace Engineering, Delft University of Technology, Kluyverweg 1, 2629 HS Delft, The Netherlands; 2 Department of Space Systems Engineering, Faculty of Aerospace Engineering, Delft University of Technology, Kluyverweg 1, 2629 HS Delft, The Netherlands

**Keywords:** Relative localization, Collision avoidance, Micro Air Vehicles, Autonomous flight, Indoor exploration

## Abstract

To avoid collisions, Micro Air Vehicles (MAVs) flying in teams require estimates of their relative locations, preferably with minimal mass and processing burden. We present a relative localization method where MAVs need only to communicate with each other using their wireless transceiver. The MAVs exchange on-board states (velocity, height, orientation) while the signal strength indicates range. Fusing these quantities provides a relative location estimate. We used this for collision avoidance in tight areas, testing with up to three AR.Drones in a $4\,\mathrm{m}~\mathbf {\times }~4\,\mathrm{m}$ area and with two miniature drones ($\approx 50\,\mathrm{g}$) in a $2~\mathrm{m}~\mathbf {\times }~2~\mathrm{m}$ area. The MAVs could localize each other and fly several minutes without collisions. In our implementation, MAVs communicated using Bluetooth antennas. The results were robust to the high noise and disturbances in signal strength. They could improve further by using transceivers with more accurate signal strength readings.

## Introduction

The agility and small scale of Micro Air Vehicles (MAVs) make them ideal for indoor exploration (Kumar and Michael 2012). We imagine several autonomous MAVs navigating through a building for mapping or inspection. The agents could spread out and thus complete the exploration task in a short time. This approach also brings robustness, scalability, and flexibility to the system, being no longer tied to the success and abilities of one unit (Brambilla et al. 2013). During this scenario, however, it may happen that a few MAVs end up flying together in a small area (e.g., an office, meeting room, or hallway), leading to a high risk of inter-MAV collisions (Szabo 2015). This is a failure condition to be avoided to ensure mission success without the unwanted loss of units. We have developed and tested a method to tackle this issue which uses only decentralized wireless communication between MAVs. Two or more MAVs estimate their relative location via the wireless connection and adjust their path to avoid collisions. In this paper, we describe the details of the algorithm and present real-world results on autonomous MAVs.

The primary contribution in this article is an on-board relative localization method for MAVs based on inter-MAV wireless communication. The communication channel is used as a method for the exchange of own state measurements and as a measure of relative range (based on signal strength), providing each MAV with sufficient data to estimate the relative location of another. Our implementation uses Bluetooth, which is readily available at a low mass, power, and cost penalty even on smaller MAVs (McGuire et al. 2016). The advantages of our solution are: (a) it provides direct MAV-to-MAV relative location estimates at all relative bearings; (b) it does not require any external system such as beacons; (c) it does not require knowledge of global positions; (d) it does not depend on the lighting and sound conditions of the environment; (e) it has low mass, battery, and processing requirements; (f) it does not require dedicated sensors. Our solution also applies to other indoor localization applications, because it shows that only one access point is sufficient to obtain a localization estimate, as opposed to multiple ones as in current state of the art (Malyavej et al. 2013; Choudhry et al. 2017). The system can also be implemented on teams with more than two agents. This can be done by setting up multiple MAV-to-MAV parallel instances of the estimator.

The secondary contribution in this article is a reactive collision avoidance strategy that is designed to deal with the localization estimator. The strategy is based on the concept of collision cones (Fiorini and Shiller 1998), tailored to suit the relative localization method and its expected performance.

The paper is organized as follows. First, we review related literature in Sect. 2. Then, Sect. 3 introduces the relative localization method and Sect. 4 describes our collision avoidance strategy. To assess the system, we developed a representative room exploration task, explained in Sect. 5. We started with simulation trials to test the system under different conditions (Sect. 6). Then, the technology was implemented on AR.Drones. Initially, the AR.Drones were aided by using ego-motion data from an external tracking system, as detailed in Sect. 7. This was done to isolate the performance of the relative localization from other sensors. Next, this was removed so that the AR.Drones were relying on on-board sensors, for which the set-up and results can be found in Sect. 8. Finally, the system was implemented on miniaturized MAVs (Sect. 9). All results are further discussed in Sect. 10. Concluding statements and future challenges are laid out in Sect. 11.

## Related work and research context

MAVs need to be as efficient as possible so as to decrease mass and maximize flight time. This means that they are often limited in sensing, computational power, and payload capabilities (Remes et al. 2014; Mulgaonkar et al. 2015). Inter-MAV collision avoidance is important for mission success but it must not exhaust the already limited resources, which should remain free to pursue the real mission. Arguably, the simplest method to avoid collisions is to have the MAVs fly at different heights. However, experiments by Powers et al. (2013) have shown that MAV multi-rotors flying over each other experience and/or cause considerable aerodynamic disturbances. Furthermore, height sensor (e.g., sonar) readings could be disturbed. Based on this limitation, we conclude that lateral evasive maneuvers are needed, and these require relative location estimates between MAVs.

One method to achieve relative localization is to provide a shared reference frame in which each MAV knows its own absolute location. The MAVs can share absolute position data and infer a relative estimate. In outdoor tasks, Global Navigation Satellite System (GNSS) receivers can be used to obtain global position data to share. This has enabled formation flying (Min et al. 2016) and large-scale flocking (Vásárhelyi et al. 2014). In indoor tasks, where GNSS is not available, absolute position data can be measured using external sensors/beacons in a known configuration, such as: motion tracking cameras (Michael et al. 2010), fixed wireless transmitters/receivers (Guo et al. 2016; Ledergerber et al. 2015), or visual markers (Faigl et al. 2013). However, these solutions are unsuitable for exploration tasks of unknown and unstructured environments. Simultaneous Localization and Mapping (SLAM) methods circumvent this by generating a map on-board during flight, which then provides position information that can be shared (Scaramuzza et al. 2014). However, if on-board map generation is not part of the mission then this is a resource intensive practice to be discouraged (Ho et al. 2015). Therefore, the more direct strategy is for the MAVs to directly localize each other.

Vision has received significant attention as a method for direct localization, where front-facing cameras are used to detect and localize other MAVs. Current implementations generally adopt mounted visual aids in the form of: colored balls (Roelofsen et al. 2015), tags (Conroy et al. 2014), or markers (Nägeli et al. 2014). However, experiments during exploratory phases of this study have shown that the use of vision without such aids, for very small drones, and at low resolution [$128\, \mathrm{px} \times 96\,\mathrm{px}$, as seen on a Lisa-S Ladybird (McGuire et al. 2016)], is prone to either false positives or false negatives. Other disadvantages of using vision are: dependence on lighting conditions, the need for a front-facing camera, limited field-of-view, and high processing requirements (Alvarez et al. 2016).

Roberts et al. (2012) proposed using Infra-Red (IR) sensors. If arranged in an array, this enables an accurate measure of relative bearing between two MAVs. Unfortunately, because IR is uni-directional, several sensors are needed to each face in a specific direction. This is not easily portable to smaller MAVs.

Alternatively, recent work by Basiri (2015) uses on-board sound-based localization. A microphone array and a chirp generator are mounted on-board of the MAVs, and the difference between arrival times of the chirp at the different microphones is used to estimate the relative bearing (Basiri et al. 2014, 2016). This method requires dedicated hardware, which for smaller MAVs can account for an increase in mass of even 10–20% (Basiri et al. 2016; Remes et al. 2014).

To truly minimize the footprint, we focused on a component that is mounted by necessity on all MAVs: a wireless transceiver. This is typically used for communication with a ground station (Lehnert and Corke 2013; McGuire et al. 2016), but it may also be used for communication between the MAVs. The signal strength of a wireless communication decreases with distance from the antenna, and can be used as a measure for range between MAVs. Signal strength ranging has been used to obtain relative localization between modules using Multi-Dimensional Scaling (MDS) (Li et al. 2007). Unfortunately, these methods need more than two agents to function, whereas we are equally interested in avoiding a simple collision between two MAVs. In previous work by our group at the Micro Air Vehicle Laboratory, we first exploited signal strength on-board of real MAVs for collision avoidance (Szabo 2015). However, range-only measurements, coupled with significant noise and disturbances, were found insufficient to guarantee safe flight of two or more MAVs in a confined area despite using a complex evolved avoidance behavior. Lijina and Nippun Kumaar (2016) recently also explored wireless-range avoidance on WeBot robots (in simulation only), but range measurements were aided by an array of proximity sensors.

Transceivers can be exploited for both ranging and data exchange. Based on this, we developed a fusion filter to estimate relative location using range and the communicated states between the MAVs. To the best of our knowledge, the only instance of on-board relative localization using a wireless transceiver was recently brought forward by Guo et al. (2017) with Ultra Wide-Band (UWB) technology. However, they make use of one of the MAVs as a beacon and their method relies on highly accurate distance measurements. Instead, we propose a method that complements possibly noisy distance measurements by communicating on-board states between moving MAVs. We then show how it can be used for indoor collision avoidance. We extensively validate this on real platforms as light as 50 g that communicate between each other using Bluetooth, which is highly prone to noise and disturbances.

## Communication-based relative localization

Relative localization is achieved via wireless communication between the MAVs. The MAVs communicate the following states to each other: planar velocity in the body frame, orientation with respect to North, and height from the ground. When communicating, the MAVs can also measure the signal strength; this acts as a measure of distance. For Bluetooth Low Energy (BLE), the technology chosen in our implementation, signal strength measurements are referred to as Received Signal Strength Indication (RSSI). Each MAV fuses the received states, the RSSI, and its own on-board states to estimate the relative position of another MAV. When multiple MAVs are present, each MAV can run multiple parallel instances of the fusion filter so as to keep track of all others. This section details the design and implementation of the relative localization scheme and presents some preliminary localization results that were obtained in early stages of the research.

### Framework definition for relative localization

**Fig. 1:**
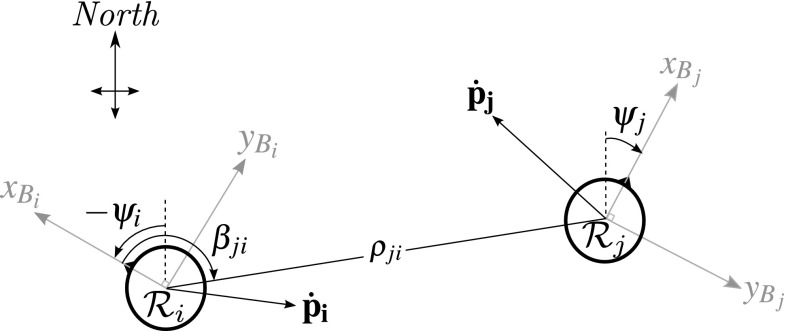
Top view of the relative localization framework ($x_B$ and $y_B$ are the planar axis of ${\mathcal {F}}_{B}$, while $z_B$ is positive down)

Consider two MAVs ${\mathcal {R}}_i$ and ${\mathcal {R}}_j$ with body-fixed frames ${\mathcal {F}}_{{B}_i}$ and ${{\mathcal {F}}}_{{B}_j}$, respectively. We define the relative pose of ${\mathcal {R}}_j$ with respect to ${\mathcal {R}}_i$ as the set ${{P}}_{ji} = \{ \rho _{ji}, \beta _{ji}, z_{ji}, \psi _{ji}\}$, where $\rho _{ji}$ represents the range between the origins of ${\mathcal {F}}_{{B}_i}$ and ${\mathcal {F}}_{{B}_j}$, $\beta _{ji}$ is the horizontal planar bearing of the origin of ${\mathcal {F}}_{{B}_j}$ with respect to ${\mathcal {F}}_{{B}_i}$, $z_{ji}$ is the height of ${\mathcal {R}}_j$ with respect to ${\mathcal {R}}_i$ and $\psi _{ji}$ is the yaw of ${\mathcal {F}}_j$ with respect to ${\mathcal {F}}_i$. See Fig. 1 for an illustration. Note that $\rho _{ji}$ and $\beta _{ji}$ are related to their Cartesian counterparts via:

1 $$\begin{aligned} \rho _{ji}&= \sqrt{x_{ji}^2 + y_{ji}^2 + z_{ji}^2}, \end{aligned}$$

2 $$\begin{aligned} \beta _{ji}&= atan2(y_{ji}, x_{ji}). \end{aligned}$$

$x_{ji}$, $y_{ji}$, and $z_{ji}$ are the Cartesian coordinates of the origin of ${\mathcal {R}}_j$ in ${\mathcal {F}}_{{B}_i}$.

### Signal strength as a range measurement

**Fig. 2:**
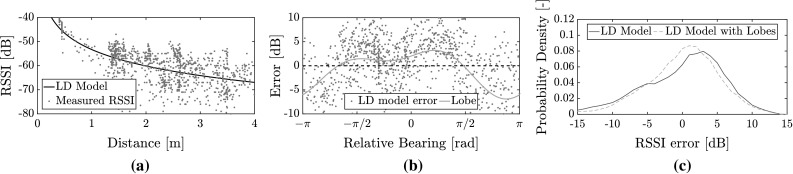
Results of RSSI measurements during an experiment whereby a Ladybird MAV was carried in circles around a fixed Bluetooth antenna. **a** RSSI measurements with respect to distance and fitted LD model, **b** error about LD model with respect to relative bearing fitted with a second order Fourier series, **c** noise distribution about the LD model without and with lobe effects

Let $S_{ji}$ be the RSSI measurement in dB. It is correlated with $\rho _{ji}$ by a function $\mathcal {L}(\rho _{ji})$. We define this function based on the Log-Distance (LD) model (Seybold 2005):

3 $$\begin{aligned} S_{ji} = \mathcal {L}(\rho _{ji}) = P_n - 10 * \gamma _{l} * \log _{10}(\rho _{ji}). \end{aligned}$$

$P_n$ is the RSSI at a nominal distance of 1 m. $\gamma _{l}$ is the *space-loss parameter*, which dictates how much the signal strength decays with distance (for free-space: $\gamma _{l} = 2.0$).^1^ The LD model is assumed subject to Gaussian noise (Svečko et al. 2015).

In preliminary tests, we analyzed the LD model with a Ladybird MAV (Remes et al. 2014) connected via Bluetooth to a fixed W1049B omni-directional antenna (Pulse 2008). The MAV was carried in concentric circles at different distances around the antenna whilst RSSI was being recorded with the antenna. The orientation of the MAV with respect to North was kept constant, thus varying the relative bearing to the antenna. Ground-Truth (GT) data was recorded with an Optitrack Motion Capture System (MCS). The results from a representative data sample are shown in Fig. 2, to which the LD model was fitted using a non-linear least squares estimator as in Fig. 2a. Among a set of similar experiments, the Standard Deviation (SD) of the error about the fitted LD model was found to be between 3 and 6 dB. This is in line with literature (Szabo 2015; Nguyen and Luo 2013).

We also observed a change of the error with the relative bearing. This is shown in Fig. 2b, and accounts for the skew in error distributions, see Fig. 2c. The disturbances that can this are uneven directional propagation lobes, interference by the reflection of the signal in the environment, the presence of other signals in the 2.4 GHz spectrum, or other objects that obstruct the signal (Seybold 2005; Svečko et al. 2015; Szabo 2015; Kushki et al. 2008; Caron et al. 2008). Such disturbances could be dependent on the environment or on the relative bearing between antennas, both of which are unknown during an exploration task. For this reason, the LD model was not expanded to include this dependency on bearing.

### Localization via fusion of range and on-board states

Achieving a relative pose estimate requires measuring or inferring all four variables in ${P}_{{ji}}$. We can directly measure or observe the following three:

- $\rho _{ji}$ (range), available via RSSI as in Sect. 3.2.
- $z_{ji}$ (relative height). Each MAV is expected to measure its height above the ground. This could be done with a pressure sensor (Beard 2007; Sabatini and Genovese 2013; Shilov 2014), sonar, or a downward-facing camera (Kendoul et al. 2009a, b). Two MAVs ${\mathcal {R}}_i$ and $\mathcal {R}_j$ can share their altitude data, such that: $z_{ji} = z_j - z_i$.
- $\psi _{ji}$ (relative orientation). It is assumed that all MAVs acknowledge a common planar axis [e.g., magnetic North (No et al. 2015; Afzal et al. 2011)]. Through communication, the MAVs exchange their orientation data.

Relative bearing is the only unknown variable. It becomes observable when fusing the three measurements above with velocity measurements (Martinelli and Siegwart 2005; Martinelli et al. 2005).^2^ We chose to perform sensor fusion with a discrete-time Extended Kalman Filter (EKF) due to its efficient processing and memory requirements (De Silva et al. 2014). The filter uses Cartesian coordinates so that it can directly take the difference between velocities in each axis. The state transition model from time-step *k* to $k+1$ was defined as in Eq. (4).

4 $$\begin{aligned} \begin{bmatrix} \mathbf {p}_{ji} \\ \dot{\mathbf {p}}_{i} \\ \dot{\mathbf {p}}_{jRi} \\ \psi _{j} \\ \psi _{i} \\ z_{j} \\ z_{i} \end{bmatrix}_{k+1}&= \begin{bmatrix} \mathbf {p}_{ji} + \left( \dot{\mathbf {p}}_{jRi} - {\dot{\mathbf {p}}_{i}}\right) \varDelta t \\ \dot{\mathbf {p}}_{i} \\ \dot{\mathbf {p}}_{jRi} \\ \psi _{j} \\ \psi _{i} \\ z_{j} \\ z_{i} \end{bmatrix}_k + \mathbf {v}_k \end{aligned}$$

$$\mathbf {p}_{ji} = [\begin{array}{ll} x_{ji}&y_{ji}] \end{array}^T$$ holds Cartesian equivalents of relative bearing and range. $$\dot{\mathbf {p}}_i = [\begin{array}{ll} \dot{x}_{i}&\dot{y}_{i} \end{array}]^T$$ is a vector of the velocity of ${\mathcal {R}}_i$ in ${\mathcal {F}}_{{B}_i}$ (see Fig. 1). $\dot{\mathbf {p}}_{jRi}$ is $\dot{\mathbf {p}}_j$ rotated from ${\mathcal {F}}_{{B}_j}$ to ${\mathcal {F}}_{{B}_i}$. $\varDelta t$ is a discrete time-step between updates, equal to the time between *k* and $k+1$. $\mathbf {v}_k$ represents the noise in the process at time-step *k*. This model assumes that all current velocities and orientations remain constant between time-steps. The observation model for the EKF is given by Eq. (5).

5 $$\begin{aligned} \begin{bmatrix} S_{ji} \\ \dot{\mathbf {p}}_{i} \\ \dot{\mathbf {p}}_{j} \\ \psi _{j} \\ \psi _{i} \\ z_j \\ z_i \end{bmatrix}_{k}&= \begin{bmatrix} {\mathcal {L}}(\rho _{ji}) \\ \dot{\mathbf {p}}_{i} \\ {\mathbf {R}}_{\mathbf {2D}}\varvec{(\psi }_{\mathbf {ji}}\varvec{)} * \dot{\mathbf {p}}_{jRi} \\ \psi _{j} \\ \psi _{i} \\ z_j \\ z_i \end{bmatrix}_k + \mathbf {w}_k \end{aligned}$$

$\mathbf {R_{2D}({\cdot })}$ is a 2D rotation matrix that uses the relative heading $\psi _{ji}$ to rotate the state estimate $\dot{\mathbf {p}}_{jRi}$ from ${\mathcal {F}}_{B_i}$ to ${\mathcal {F}}_{B_j}$. $\mathbf {w}_k$ represents the noise in the measurements at time-step *k*. Note that $\rho _{ji}$ is expanded as per Eq. (1) so as to observe $x_{ji}$ and $y_{ji}$. The EKF cannot be initialized with a correct relative location estimate, since this is not known; it must converge towards the correct value during flight. Appropriate tuning of the EKF noise covariance matrices is key to achieving this. In the EKF, the measurement noise matrix $\mathbf {R}$ is a diagonal matrix with the form shown in Eq. (6).6$\sigma _{m}$ is the assumed SD of $S_{ji}$. $\sigma _{v}$ is the assumed SD of $\dot{\mathbf {p}}_i$ and $\dot{\mathbf {p}}_j$. $\sigma _{\psi }$ is the assumed SD of the magnetic orientation measurements. $\sigma _{z}$ is the assumed SD of the height measurements. $\mathbf {I_{n\times n}}$ is a $n\times n$ identity matrix. Based on our preliminary RSSI noise analysis, $\sigma _{m}$ is tuned to 5 dB. Throughout this paper, all other SDs were tuned to 0.2, unless otherwise stated. This was based on the measurement noise, either simulated or expected from the sensors.

The process noise matrix $\mathbf {Q}$ is the diagonal matrix presented in Eq. (7).7$\sigma _{{Q}_{p}}$ is the SD of the process noise on the relative position update. $\sigma _{{Q}_{v}}$, $\sigma _{{Q}_{\psi }}$, and $\sigma _{{Q}_{z}}$ are SDs for the expected updates in velocity, orientation, and height, respectively. By tuning $\mathbf {Q}$ we can define the validity of the process equations (Malyavej et al. 2013). In this paper, unless otherwise stated: $\sigma _{{Q}_{p}} = 0.1$, while $\sigma _{{Q}_{v}} = \sigma _{{Q}_{\psi }} = \sigma _{{Q}_{z}} = 0.5$. We tuned $\sigma _{{Q}_{p}}$ to 0.1 so as to have a relatively low process noise on the relative position update. This forces the filter to rely less on the (noisy) range measurements and more on other data, which encourages convergence and helps discard the high noise and disturbance in the RSSI measurements. $\sigma _{{Q}_{v}}$, $\sigma _{{Q}_{\psi }}$, and $\sigma _{{Q}_{z}}$ were then tuned higher (to 0.5) to enhance the difference, while staying within the order of magnitude of the expected standard deviations of the measurements.

**Fig. 3:**
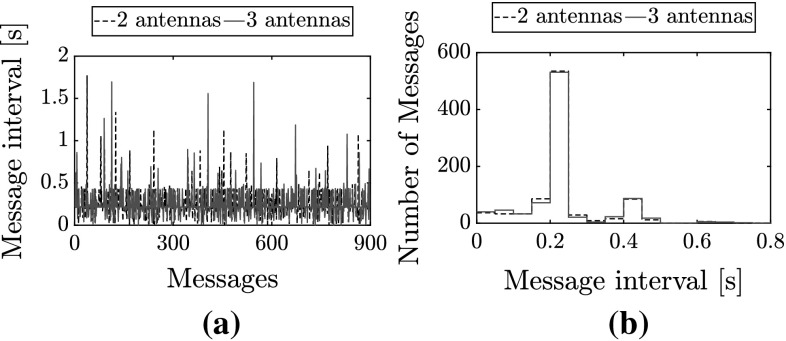
Messaging rate over a test flight. **a** Message intervals, **b** distribution

This filter is limited by flip and rotation ambiguity as defined by Cornejo and Nagpal (2015). When the motion of ${\mathcal {R}}_j$ perfectly matches the motion of ${\mathcal {R}}_i$, range-only measurements remain constant and are not informative for bearing estimation. Unless the MAVs are flying in formation, the probability of this event will be low (Cornejo and Nagpal 2015). The same ambiguity takes place when both ${\mathcal {R}}_i$ and $\mathcal {R}_j$ are static. Motion by at least one MAV is required, as the filter operates by taking the difference in velocity. The performance of the filter thus increases as the average difference in velocity between the MAVs increases (and/or the accompanying measurement noise decreases).

**Fig. 4:**
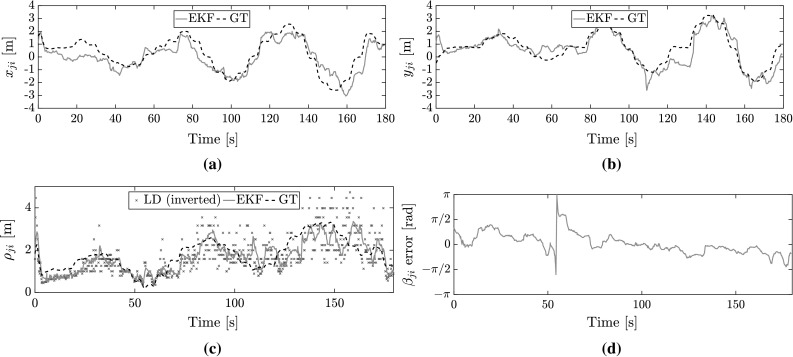
Preliminary localization trial based on circular flights of a Ladybird MAV around a fixed antenna (with artificial noise added to the velocity, height, and orientation measurements). **a** Ground-truth versus estimated location of the MAV along the $x_B$ axis of the antenna. **b** Ground-truth versus estimated location of the MAV along the $y_B$ axis of the antenna. **c** Comparison of EKF estimated range compared to ground-truth and estimate from inverting the LD model. **d** Error in $\beta _{ji}$ over time

### Implementation details

We used BLE to enable communication between the MAVs. The data is sent and received by means of advertising messages scheduled using a Self-Organized Time Division Multiple Access (STDMA) algorithm, as described by Gaugel et al. (2013). This enables ad-hoc communication and circumvents the Master-Slave paradigm otherwise enforced by the BLE standard (Townsend et al. 2014), as each antenna alternates between advertising and listening. The messaging rate is tuned to 5 Hz, which is a compromise between the amount of STDMA communication slots (8 slots) and an acceptable communication rate. 5 Hz keeps the congestion low. Nevertheless, the messaging rate can be affected by differences in clock rates and possible packet losses. Figure 3 shows the interval between received messages rate over approximately 3 min of recording from the point of view of a single antenna in a group of two or three participating antennas. Approximately 80% of messages are received and parsed within 0.25 s and 95% within 0.45 s. A slight increase in packet loss was observed when increasing the number of antennas to three, with 128 missed messages as opposed to 120 when two antennas were used. This corresponds to an increase in packet loss from 13.4 to 14.2%.

### Preliminary relative localization tests

We performed preliminary localization tests with a Ladybird MAV flying around a fixed Bluetooth W1049B antenna. The objective was to determine how well the antenna could localize the MAV. An Optitrack MCS was used to guide the MAV in circular flights and record its GT velocity, orientation, and height. The antenna measured the RSSI to the MAV. The recorded GT data was altered with Gaussian noise with $\sigma _{v} = 0.2$ m/s, $\sigma _{z} = 0.2$ m, and $\sigma _{\psi } = 0.2$ rad, and then used as measurements for the EKF. In the LD model of the EKF: $P_n = -\,63$ dB and $\gamma _l = 2.0$. The EKF was initialized with a null guess position of $x_{ji}=y_{ji}=1$ m. In these preliminary tests, the localization filter was applied off-board.

Estimates for $x_{ji}$ and $y_{ji}$ are shown in Fig. 4a, b, the EKF converges towards GT in the first few seconds, after which it tracks successfully. The small oscillations in the GT are induced by the fact that the MAV did not travel continuously, but via way-point navigation. Figure 4c shows the estimated range, where we can observe a significant improvement in error with respect to an inverted LD model. Note that the range error increases with distance, this is due to the logarithmic nature of RSSI propagation. Figure 4d shows the bearing error, which is small throughout most of the flight. A noticeable exception is a spike about the 55 s mark. This is because, at very small distances, a small error in $x_{ji}$ or $y_{ji}$ can translate into a large error in $\beta _{ji}$. It can reach $\pm \,\pi$ if $x_{ji}$ or $y_{ji}$ estimates are both of the wrong sign, as it briefly happens at around 55 s.

## Collision avoidance behavior

The avoidance algorithm is based on the Collision Cone (CC) framework (Fiorini and Shiller 1998; Wilkie et al. 2009). A collision cone is a set of all velocities of an agent that are expected to lead to a collision with an obstacle at a given point in time. Its name derives from the fact that it is geometrically cone-shaped. In our work, we scale the collision cones according to the expected relative localization errors and implement a clockwise search to find escape directions not covered by cones. Using this framework bears these main advantages: (a) the MAVs are not encouraged to vary their speed (slow down), as motion is beneficial to the performance of the relative localization filter (see Sect. 3.3), and also select a predictable escape direction. This is different to Velocity Obstacle (VO) approaches (Wilkie et al. 2009; b) there can be an explicit relationship between the size of the cone and the expected localization error; (c) distance, which has a low estimation accuracy, is not used to make decisions. This differentiates it from distance-based methods such as repulsion forces (Virágh et al. 2016). This section details our implementation of collision cones and how they are used to determine a collision-free trajectory.

**Fig. 5:**
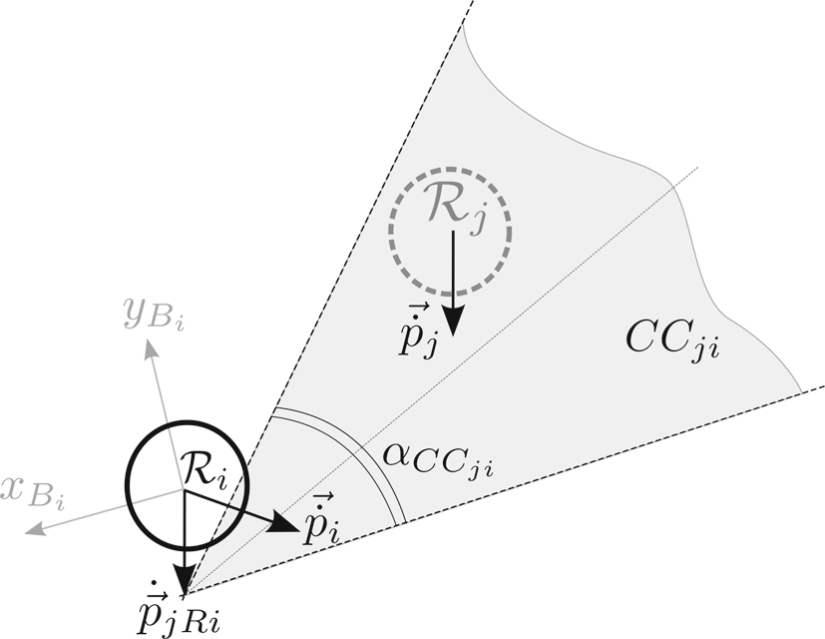
Depiction of $CC_{ji}$ that ${\mathcal {R}}_i$ holds with respect to the estimated location of ${\mathcal {R}}_j$

### Collision cones and avoidance strategy

Take two MAVs ${\mathcal {R}}_i$ and $\mathcal {R}_j$. The collision cone $CC_{ji}$, depicted in Fig. 5, would include all velocities of ${\mathcal {R}}_i$ which could lead to a collision with ${\mathcal {R}}_j$. It is constructed in three steps.

1. A cone $CC_{ji}$ is defined as in Eq. (8). $\alpha$ is an arbitrary angle. *x* and *y* are points on $x_{B_i}$ and $y_{B_i}$, respectively. The cone is characterized by an expansion angle $\alpha _{{CC}_{ji}}$, subject to $0<\alpha _{{CC}_{ji}}<\pi$.
  8 $$\begin{aligned}&CC_{ji}\nonumber \\&\quad = \left\{ (x,y) \in {\mathbb {R}}^2;\; {\alpha } \in {\mathbb {R}};\; {|}{\alpha }{|}{\le }\frac{|{\alpha _{{CC}_{ji}}}|}{2}:\tan (\alpha )x = y\right\} \end{aligned}$$
2. $CC_{ji}$ is rotated so as to be centered around the estimated bearing to the obstacle ${\mathcal {R}}_j$ as in Eq. (9), where: $\bar{\beta }_{ji}$ is the estimated ${\beta }_{ji}$ from the EKF, $\leftarrow$ is an update operator, and $\mathbf {R}(\cdot )$ is a rotation operator for the set.
  9 $$\begin{aligned} {CC}_{ji} \leftarrow \left( \mathbf {R}(\bar{{\beta }}_{ji}) * {CC}_{ji} \right) \end{aligned}$$
3. The cone is translated by the estimated velocity of ${\mathcal {R}}_j$ expressed in ${\mathcal {F}}_{B_i}$, as per Eq. (10). This accounts for the fact that the obstacle is moving. $\overline{\dot{\mathbf {p}}_{jRi}}$ is the estimated $\dot{\mathbf {p}}_{jRi}$ from the EKF. The operator $\oplus$ denotes the translation of a set by a vector.
  10 $$\begin{aligned} {CC}_{ji} \leftarrow {CC}_{ji} \oplus \overline{\dot{\mathbf {p}}_{jRi}} \end{aligned}$$

In a team of *m* MAVs, each member ${\mathcal {R}}_i$ holds $m-1$ collision cones that it can superimpose into a single set ${CC}_i$.

11 $$\begin{aligned} {CC}_i =\bigcup \limits _{j=1}^{m-1} {CC}_{ji} \end{aligned}$$

If, during flight, ${\dot{\mathbf {p}}_i} \in {CC}_i$, then a clockwise search about the $z_{B_i}$ axis (starting with the current desired velocity) is used to determine the desired escape velocity. If no solution is found, then the search is repeated for a higher escape speed.^3^

A clockwise search encourages a preference for right-sided maneuvers with respect to the current flight direction. This differentiates it from the VO avoidance method, which selects a flight direction that minimizes the required change in velocity. Although effective, when an agent opts for the minimum change in velocity without considering that the other might also change its motion, this may lead to an issue known as *“reciprocal dances”*. These happen when two entities heading towards each other repeatedly select the same escape direction. The situation is analogous to when two people try to avoid each other in a corridor and both select the same direction, leading to a left–right dance by each person. In other literature, solutions to VO’s reciprocal dances rely on the reciprocity assumption, meaning that each agent assumes that the other agent will also take a predefined evasive action (Snape et al. 2009, 2011; Van Den Berg et al. 2011). In our case, however, due to the potential for large relative localization errors, MAVs cannot safely assume that the others will participate in a suitable and reciprocal escape maneuver. Enforcing right sided maneuvers is a solution to limit oscillations without assuming reciprocity. It should be noted, however, that oscillations may still occur when the MAVs have to coordinate between multiple obstacles, such as a wall and another MAV, or multiple MAVs.

### Tuning the expansion angle of the collision cone

The expansion angle of a collision cone is dependent on the distance between the MAVs (the MAV radii becomes more significant as distance decreases) and the relative estimation errors (Conroy et al. 2014). Based on this knowledge, we formulated Eq. (12) to calculate the expansion angle,

12 $$\begin{aligned} \alpha _{{CC}_{ji}} = 2*\tan ^{-1}{\left( \frac{2r + {\bar{\rho }}_{ji} + \varepsilon _\alpha }{\kappa _\alpha *{\bar{\rho }}_{ji}}\right) }, \end{aligned}$$

where *r* is the radius of a MAV (modeled as a circle); $\bar{\rho }_{ji}$ is the estimated range between ${\mathcal {R}}_i$ and $\mathcal {R}_j$; $\varepsilon _\alpha$ is an additional margin, the properties of which are discussed in Sect. 4.3; $\kappa _\alpha$ is a coefficient describing the quality of the estimate. The expansion angle has a lower bound $\alpha _{{CC}_{asymptote}}$ which is dependent on $\kappa _\alpha$:

13 $$\begin{aligned} \alpha _{{CC}_{asymptote}} =\lim _{\bar{\rho }_{ji} \rightarrow \infty } \alpha _{{CC}_{ji}} = 2*\tan ^{-1}{\left( \frac{1}{\kappa _\alpha }\right) }. \end{aligned}$$

**Fig. 6:**
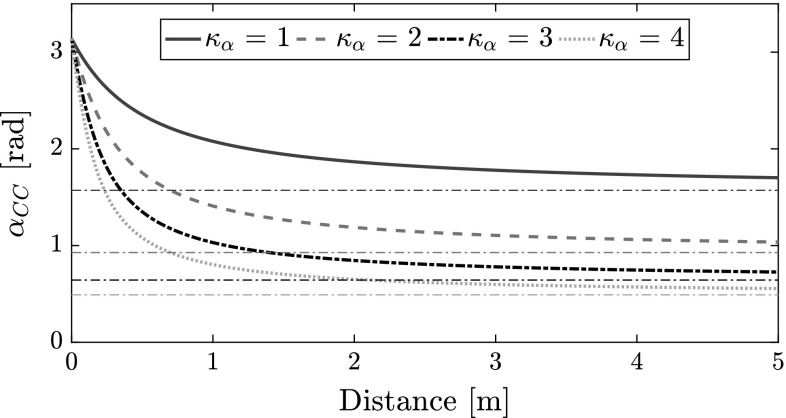
Effect of $\kappa _\alpha$ on $\alpha _{{CC}_{asymptote}}$ ($r = 0.1~\mathrm{m}$, $\varepsilon = 0.5$)

The impact of $\kappa _\alpha$ may be appreciated in Fig. 6. In this work, unless otherwise stated, we use $\kappa _\alpha =1$, so $\alpha _{{CC}_{asymptote}}=\frac{\pi }{2}$. This generally encompasses the expected bearing errors during flight based on our preliminary results.

### Preserving behavior in rooms of different size

**Fig. 7:**
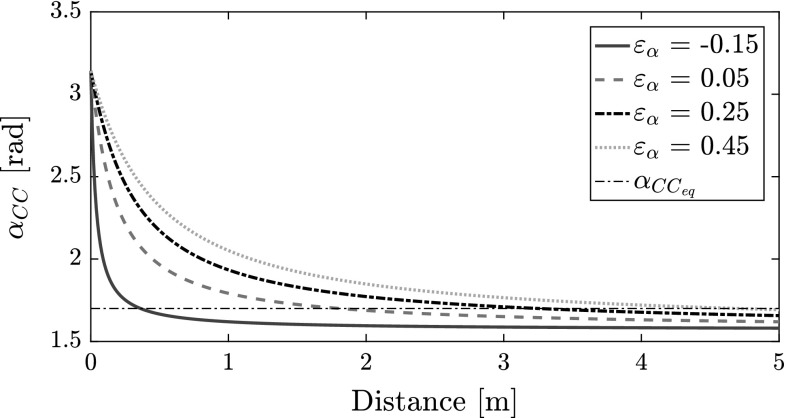
Effect of $\varepsilon _\alpha$ on $\alpha _{CC}$ ($r = 0.1~\mathrm{m}$, $\kappa _\alpha = 1$)

The expansion angle of the collision cone widens towards $\pi$ as the distance between two MAVs decreases. This implies that in smaller rooms the collision cones would *always* feature wide expansion angles, leading to most of the environment becoming out of bounds. This restriction in freedom of movement creates oscillations in MAV trajectories. To limit the issue, we propose using $\varepsilon _\alpha$ as a tuning parameter. The effect of varying $\varepsilon _\alpha$ is shown in Fig. 7; as $\varepsilon _\alpha$ decreases, the decay of the expansion angle with distance increases. A faster decay is suitable for smaller rooms so that motion is less restricted.

We devised a method to tune $\varepsilon _\alpha$ intuitively. Rearranging Eq. (12), $\varepsilon _\alpha$ is expressed by:

14 $$\begin{aligned} \varepsilon _\alpha = \kappa _\alpha * \rho _{eq} * \tan {\left( \frac{\alpha _{{CC}_{eq}}}{2}\right) } - 2r - \rho _{eq}. \end{aligned}$$

This translates tuning $\varepsilon _\alpha$ to tuning a pair $\{\alpha _{{CC}_{eq}},\rho _{eq}\}$, where $\alpha _{{CC}_{eq}}$ is the desired angle of expansion at a distance $\rho _{eq}$. Note that $\alpha _{{CC}_{eq}} > \alpha _{{CC}_{asymptote}}$, and $\varepsilon _\alpha \ge -(r_i+r_j)$ if $\kappa _\alpha \ge 1$. In all our tests, $\rho _{eq}$ is set to half of the side length of the room. $\alpha _{{CC}_{eq}}$ is kept at 1.7 rad.

## Testing methodology

An exploration task was developed where multiple MAVs fly in a room at the same altitude and attempt to pass through the center. This is designed to provoke collisions. The sample task was used to test the performance of the relative localization and collision avoidance, separately and combined. This section describes the task in more detail and outlines how it will be used for assessment.

### Description of arbitrary task for performance testing

Consider a team of *m* homogeneous MAVs. Each MAV ${\mathcal {R}}_i$ can control its velocity. Let $\dot{\mathbf {p}}_{i_{cmd,k}}$ be the desired velocity for ${\mathcal {R}}_i$ expressed in its body-frame ${\mathcal {F}}_{B_i}$ at a given time-step *k*. Let $d_{{\mathrm {wall}}_{i}}$ be the distance between ${\mathcal {R}}_i$ and the arena border that is closest to it, with $d_{\mathrm {safe}}$ being a safety distance to the arena’s borders. Note that each robot ${\mathcal {R}}_i$ features $m-1$ EKF instances to keep track of the other members and uses their outputs to determine its collision cone set $CC_i$, see Eq. (11). At each time-step *k*, the EKF outputs are updated and $CC_i$ is re-calculated. $\dot{\mathbf {p}}_{i_{cmd,k}}$ is then chosen as follows: $\dot{\mathbf {p}}_{i_{cmd,k}} = \dot{\mathbf {p}}_{i_{cmd,k-1}}$ unless conditions M1 and M2 take place.

**Fig. 8:**
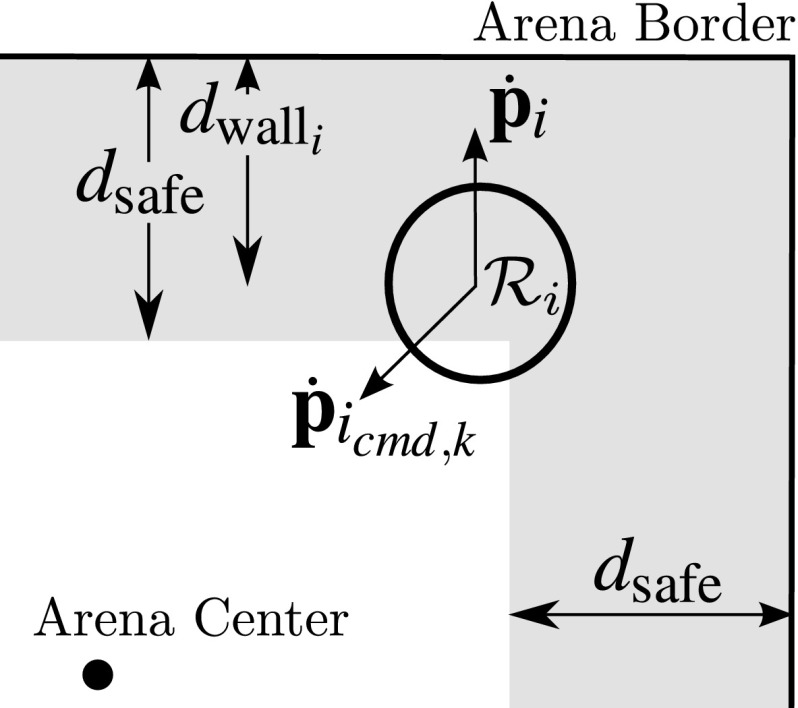
Depiction of condition M1. Robot ${\mathcal {R}}_i$ is too close to the border of the arena and receives a command to go towards the center

1. $d_{{\mathrm {wall}}_{i}} < d_{\mathrm {safe}}$ and $\dot{d}_{{\mathrm {wall}}_{i}} < 0$. This means that ${\mathcal {R}}_i$ is close to the arena border and approaching it. Then, ${\dot{\mathbf {p}}}_{i_{cmd,k}}$ is rotated towards the center of the arena as seen in Fig. 8.
2. ${\dot{\mathbf {p}}}_i \in CC_i$. This means that the current velocity of ${\mathcal {R}}_i$ could lead to a collision with one or more team members. An escape velocity is sought according to the strategy outlined in Sect. 4.

In all experiments performed in this paper, wall detection is deemed outside of our scope and is therefore enforced by using the global position of the agent within an arena (provided by a MCS). Condition M1 holds priority over M2 to ensure that the MAVs remain within the arena. At all time-steps, unless otherwise commanded by the collision avoidance algorithm, ${|}{{\dot{\mathbf {p}}}_{i_{cmd,k}}}{|}{=} v_{\mathrm {nominal}}$, where $v_{\mathrm {nominal}}$ is a fixed speed magnitude.

### Assessment strategy

Experiments were performed in stages with increasing realism and autonomy:

1. *Simulation* This enabled assessing system performance under different conditions.
2. *External own-state measurements* These tests used an external MCS to control the drones (but *not* for relative localization). This allowed to make the results independent of the ability of the drones to fly autonomously.
3. *On-board own-state measurements* These tests rely on on-board sensors to control the drones. They are used to determine real-world performance. The external MCS is only used to simulate wall detection. This type of test is performed on AR.Drones and on miniaturized MAVs. Note that for the miniaturized MAVs, the MCS was also used to measure height from the ground. This was due to the lack of a suitable height sensor on the MAVs.

The results of all tests are discussed in the next four chapters. During the tests, the three items below were assessed.

*Assessment of relative localization * The performance of the relative localization can be assessed by comparing the estimated relative locations to ground-truth data obtained cumulatively during all real-world experiments.

*Assessment of collision avoidance* This is partially dependent on the performance of the relative localization, yet can be assessed independently by identifying failure cases and observing general behavior properties. In simulation, however, it is also possible to artificially improve the relative localization estimates and establish how the collision avoidance would fare under such circumstances.

*Assessment of the full system* The parameter of interest is the mean flight time until collisions. An ideal system is one with which, systematically, collisions do not take place. Such a result may be dependent on how crowded the airspace is, so we tested different configurations. By modeling MAVs as circles, airspace density is calculated with:

15 $$\begin{aligned} \mathcal {D}_{m,c} = \frac{m * \pi r_c^2}{s_c^2} \end{aligned}$$

$\mathcal {D}_{m,c}$ denotes the density for configuration *c* with *m* MAVs, $r_c$ is the radius of a MAV in configuration *c*, and $s_c$ is the side length of the squared arena at configuration *c*.

## Simulation experiments

Simulations allow to assess the collision avoidance algorithm and the full system. We can assess the performance of the system for several airspace densities, noise scenarios, etc., and obtain statistical insights.

### Simulation environment set-up

**Fig. 9:**
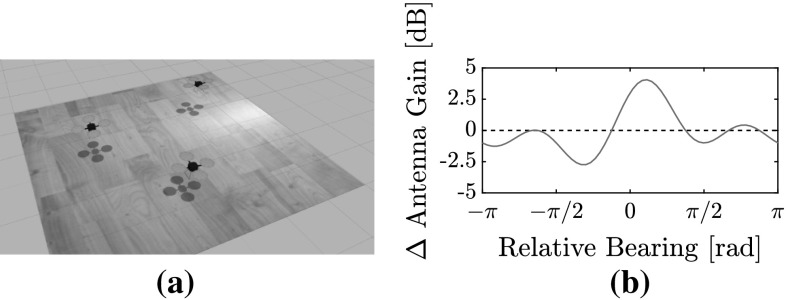
The simulation environment and the simulated lobes. **a** Screenshot of a simulation with 3 MAVs. **b** Simulated RSSI horizontal lobes applied as a function of relative bearing between MAVs

The simulation environment was developed using Robotics Operating System (ROS) (Quigley et al. 2009), the Gazebo physics engine (Koenig and Howard 2004), and the hector-quadrotor model (Meyer et al. 2012). Multiple quad-rotor MAVs can be simulated simultaneously. A ROS module (or *“node”*) for each MAV simulates Bluetooth communication and enforces the controller described in Sect. 5.1. A rendered screenshot of a simulation is shown in Fig. 9a.

The RSSI is simulated using the LD model ($P_n = -63$ dB, $\gamma _l=2.0$) with added Gaussian noise (SD of 5 dB) and horizontal antenna lobes, unless otherwise stated. The lobes were modeled using a third order Fourier series with unitary weights, see Fig. 9b. The other measurements were altered with the same standard deviations as in the preliminary localization tests of Sect. 3.5. Furthermore: $v_{\mathrm {nominal}} = 0.5$ m/s, $d_{\mathrm {safe}} = 0.25$ m, and $\psi = 0$ rad for all MAVs. The MAVs begin at different corners of the arena. The EKF is initialized such that the initial position guess is towards their initial flight direction (i.e. the center of the arena).

**Fig. 10:**
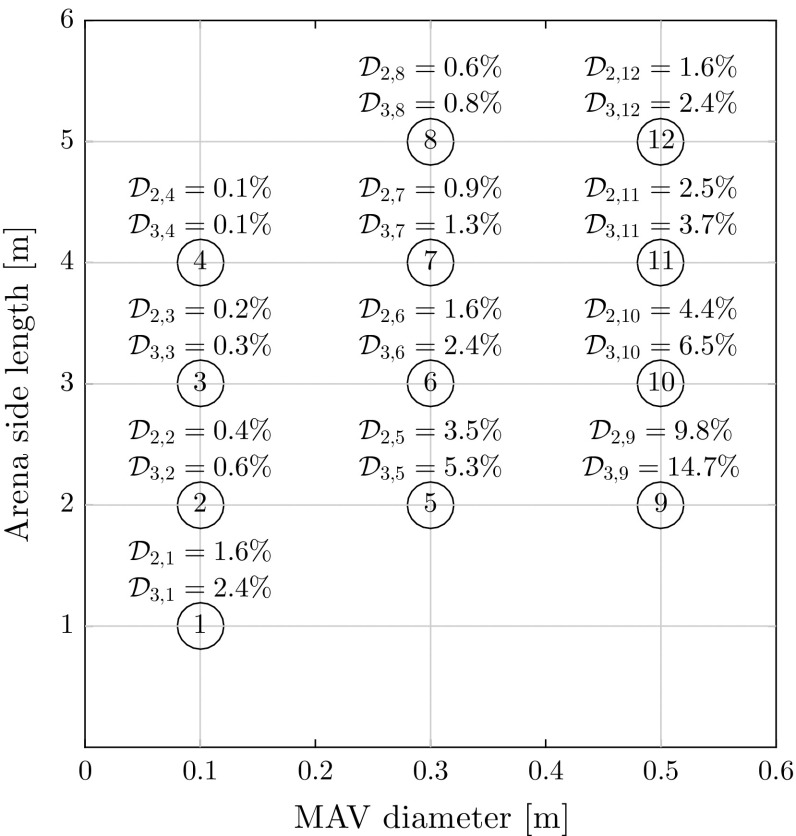
The twelve configurations tested in simulation with configuration numbers shown in the white circles. $\mathcal {D}_{m,c}$ is the airspace density for configuration *c* with *m* MAVs from Eq. (15)

We investigated twelve configurations of arena size and MAV diameter for teams of two MAVs and three MAVs. The configurations will be referred to by the encircled numbers in Fig. 10. Each configuration was simulated 100 times. Each simulation was automatically interrupted if a collision occurred or after 500 s of collision-free flight.

### Results

**Fig. 11:**
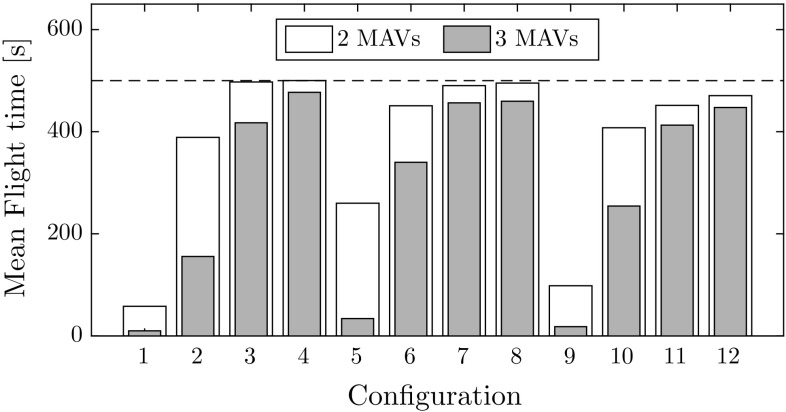
Mean flight time to collision for all simulated configurations. Average results without collision avoidance, not shown in this figure, range between 3.9 and 14.3 s

Mean flight time for each configuration is shown in Fig. 11. Flights with three MAVs consistently show a lower performance than with two MAVs. The performance drop is a result of the team dynamics at play, namely: (1) increased airspace density, and (2) decreased freedom of movement due to superposition of collision cones. These two factors are analyzed in the remainder of this section.

**Fig. 12:**
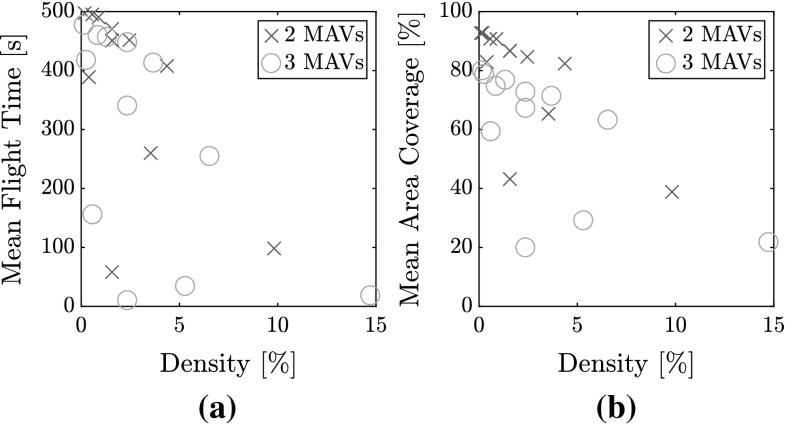
Flight parameters with respect to airspace density based on simulation results. **a** Mean flight time with respect to density. **b** Mean area coverage with respect to density

*Airspace density* When the MAV diameter remains constant, but the arena size increases, an increase in mean flight time is observed. This is seen by comparing the configuration quartets 1-2-3-4, 5-6-7-8, and 9-10-11-12. Furthermore, when the arena side-length remains constant and the MAV diameter increases, a decrease in mean flight time is observed. This is seen by comparing within the configuration triads 4-7-11, 3-6-10, and 2-5-9, and the pair 8-12. This implies that a lower density improves the probability of success, but this is found to not strictly be the case. Figure 12a shows the flight time to collision as a function of the airspace density. A portion of configurations show low results despite the low airspace density, and are outliers in the negative linear trend. These correspond to configurations 1, 2, 5, and 9, which feature smaller arena sizes. The conclusion is that room size affects performance even when airspace density remains constant. This is a remaining limitation of the current status of the system when operating in smaller rooms. Its causes are discussed in Sect. 10.2.

*Freedom of movement* Figure 12b shows the impact of airspace density on area coverage for all flights with two MAVs and three MAVs. Area coverage was measured as follows. The total area is divided in sections of $0.20~\mathrm{m}\times 0.20~\mathrm{m}$. A section is marked “covered” when one of the MAVs crosses it. Area coverage is the percentage of covered sections during a trial. Here, two patterns arise.

**Fig. 13:**
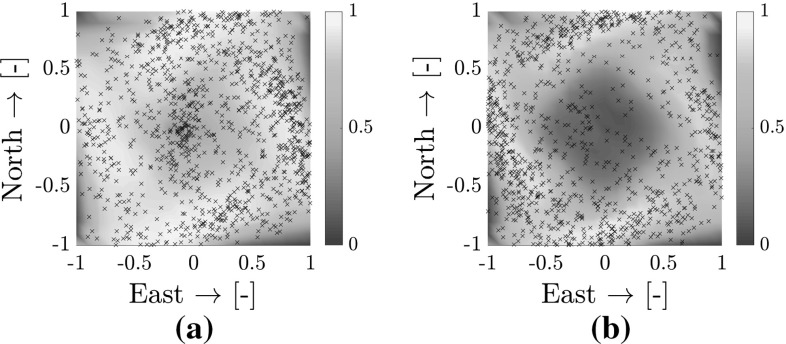
Heat maps of normalized area coverage over all simulations (black crosses indicate collisions). **a** Two MAVs. **b** Three MAVs

1. A higher airspace density leads to a lower overall coverage. This is due to: (a) lower flight times, providing less overall time to complete the mission, and (b) decreased freedom of movement due to larger portions of the arena being covered by collision cones.
2. Three MAVs systematically achieve lower area coverage than only two MAVs in the same configuration. This is explained by analyzing the flight trajectories in more detail, from which an emergent circular behavior is discerned.^4^ See Fig. 13, which shows the normalized heat map over all simulations for two (Fig. 13a) and three (Fig. 13b) MAVs. When more than one MAV to avoid is present, the superposition of multiple collision cones pushes the MAVs towards the edges.

### Impact of RSSI noise on performance

**Fig. 14:**
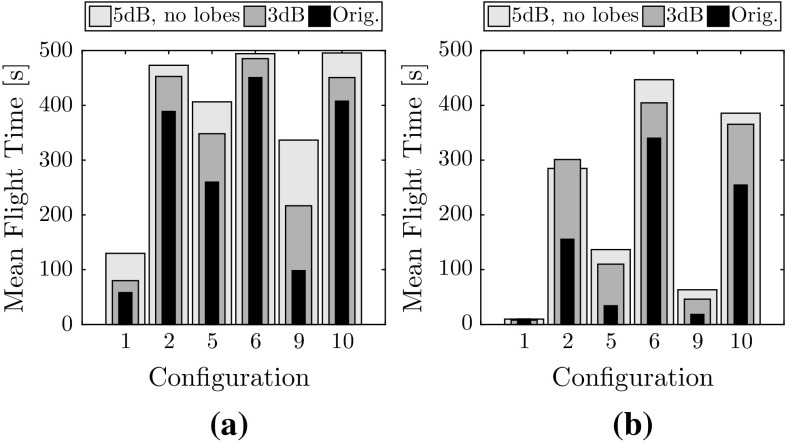
Improvements in system performance against nominal results (“Orig.”) when noise is reduced from 5 to 3 dB, or when lobes are removed. **a** Two MAVs. **b** Three MAVs

In simulation, we also tested all configuration with a clean case, where each MAV perfectly knows the position of all others. Our tests lasted 500 s (the maximum time) with no collisions. This shows that, with perfect relative localization, the collision avoidance should give perfect results. Two further case studies were explored. In the first case, the simulated RSSI noise is reduced from 5 to 3 dB, but lobes are still simulated. In the second case, RSSI noise is kept at 5 dB but sensor lobes are removed. All other parameters remain the same as in the primary simulations. The configurations tested are those with the lowest performance: 1, 2, 5, 6, 9, 10. The results are shown in Fig. 14, and show that removing the antenna lobes provides the largest improvement in performance. A lower noise also improves results, yet the impact is generally lower than antenna lobes. The lower error in relative position estimates translates into a more successful collision avoidance system. For real-world systems, this shows that performance can be improved by operating in cleaner environments, using better antennas, or by better filtering of signal strength measurements.

## Experiments featuring external own-state measurements

We implemented our system on AR.Drones. In the experiments in this section, we used Optitrack to accurately inform MAVs of their velocity, orientation, and height. This isolates the impact of using real RSSI measurements and Bluetooth communication on the relative localization system during flight. It also shows the system performance in case of high quality ego-motion estimates.

**Fig. 15:**
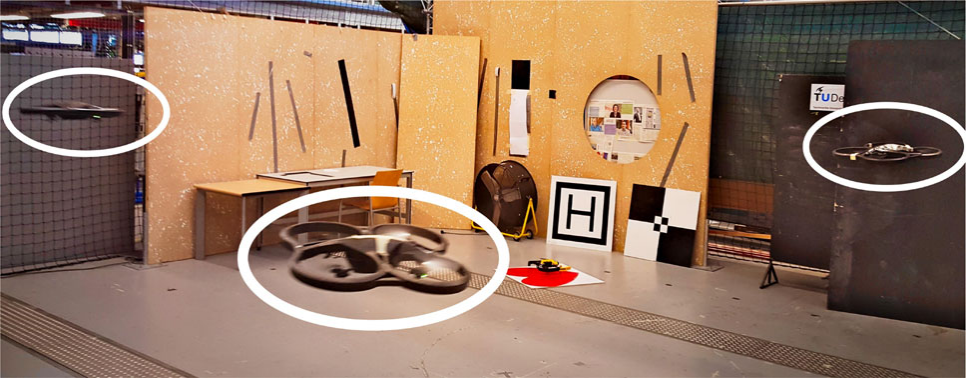
A flight with 3 AR.Drones, encircled in white

**Fig. 16:**
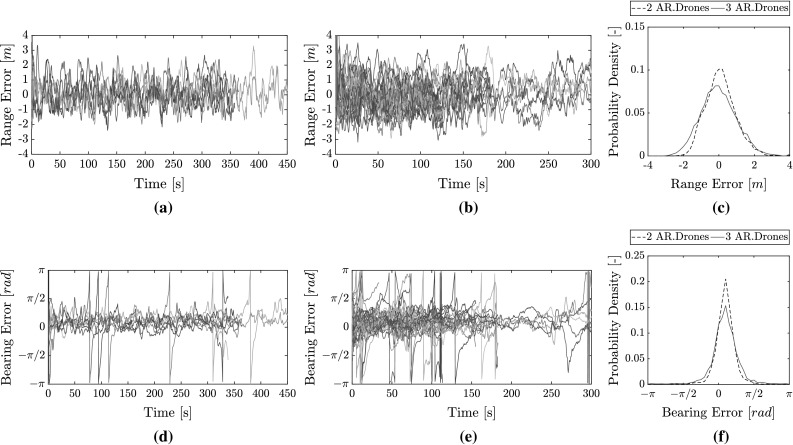
Overview of all relative range **a**, **b**, **c** and relative bearing **d**, **e**, **f** estimation errors for flights with external own-state measurements. **a** Range estimate error with two AR.Drones ($\mathrm{RMSE}=0.86\,\mathrm{m}$). **b** Range estimate error with three AR.Drones ($\mathrm{RMSE}= 1.14\,\mathrm{m}$). **c** Range error distribution for all flights. **d** Bearing estimate error with two AR.Drones ($\mathrm{RMSE} = 0.57\,\mathrm{rad}$). **e** Bearing estimate error with three AR.Drones ($\mathrm{RMSE} = 0.70\,\mathrm{rad}$). **f** Bearing error distribution for all flights

### Experimental set-up

The experiments were performed using AR.Drones 2.0 (Parrot 2012). A BLED112 (Silicon Labs 2016) Bluetooth Smart USB Dongle equipped them with Bluetooth. The controller was developed using Paparazzi (Drouin and Muller 2007) and was running entirely on-board. The experiments in this section relied on Optitrack to provide each MAV with data of its own velocity, orientation, and height via a Wi-Fi link. Each AR.Drone then communicated this data via a Bluetooth broadcast to the other ones, using the implementation described in Sect. 3.4. The Wi-Fi link was also used for take-off/land commands and for data logging. Figure 15 shows a picture of a flight with three AR.Drones.

All MAVs flew at 1.5 m from the ground, with a nominal speed $v_{\mathrm {nominal}}=0.5$ m/s and safety wall distance $d_{\mathrm {safe}}=0.5$ m. The enforced arena size in all experiments was $4~\mathrm{m}\times 4~\mathrm{m}$, making these tests analogous to configuration 11 from the simulation runs (AR.Drones are slightly larger in diameter than 0.5 m). The LD model in the EKF filter was tuned with $P_n = -\,68$ dB and $\gamma _l=2.0$. $P_n$ was obtained via a brief hand-held measurement, $\gamma _l$ was based on the free-space assumption. The Optitrack measurements inputted into the EKFs were altered with Gaussian noises $\sigma _v = 0.2$ m/s and $\sigma _\psi = 0.2$ rad.

### Results

Four flights were performed with two AR.Drones for a cumulative time of 25.3 min. Only one collision took place, which occurred in the second flight after 5.6 min. Six flights were performed with three AR.Drones for a cumulative time of 15.3 min. Five flights ended in collisions. On average, this happened after 160 s of flight.

**Fig. 17:**
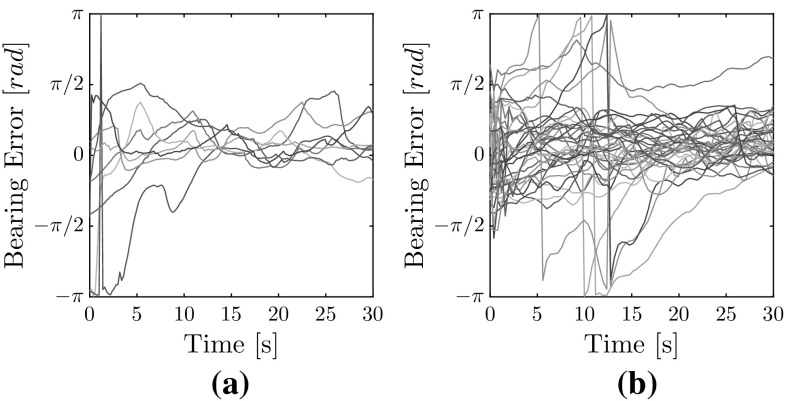
Comparison of bearing estimate errors in the first 30 s of flight during flights with external state measurements. **a** With two AR.Drones, **b** with three AR.Drones

All estimated range and bearing errors are presented in Fig. 16. The estimated range features a Root Mean Squared Error (RMSE) of 0.86 m with two MAVs and 1.14 m with three MAVs. Figure 16c shows the error distribution, indicating the lower performance with three MAVs. For bearing estimates, the Root Mean Squared Error (RMSE) for flights with two MAVs is 0.57 rad and with three MAVs it rises to 0.70 rad. Figure 16f shows the bearing error distribution. We can see the slightly larger spread of the bearing error. There is also an apparent positive bias in the error. This bias could have been caused by the initial guess of the EKF, which was unable to converge back to a zero mean. On occasion, we observe that the bearing error temporarily diverges towards $\pm \,\pi$. This error does not necessarily lead to collisions due to the non-reciprocal nature of the avoidance behavior. Nevertheless, it introduces a temporary uncertainty in the system. The error is more frequent with three AR.Drones. We also observe that the convergence rate for bearing estimates over flights with three AR.Drones appears worse than with two AR.Drones. This may be seen in Fig. 17, which zooms into the first 30 s of Fig. 16d, e. Convergence times for flights with three MAVs reach up to 30 s prior to settling (Fig. 17b). By comparison, the convergence time for flights with two AR.Drones only (Fig. 17a) is within 10 s.

**Fig. 18:**
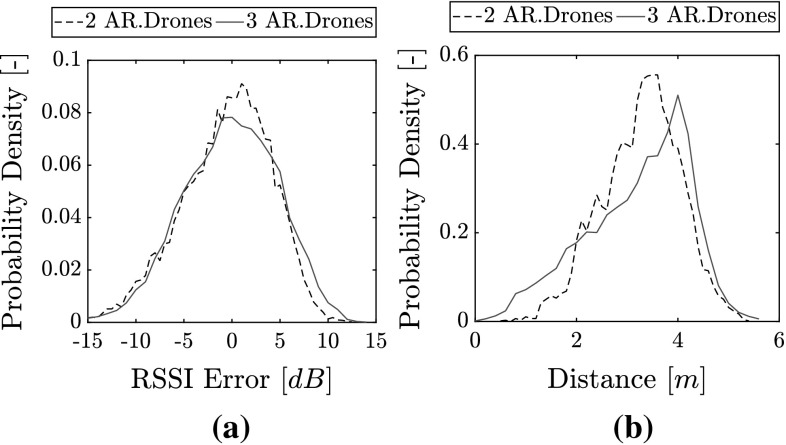
Distributions of RSSI error and distance measurements for all flights. **a** RSSI error distribution, **b** distribution of distances between MAVs based on ground-truth measurements

An analysis of the noise in RSSI showed that flights with three MAVs experienced a marginally larger noise. This can be evaluated in Fig. 18a. A two-sample Kolmogorov-Smirnov test rejected that the two distributions could be the same (with a *p* value of $6.9 \times 10^{-22}$). We also ran a bootstrap test with 10,000 repetitions, testing for the difference in sample mean and the ratio of variances as reference statistics. In both cases, the bootstrap test rejected the null hypothesis over the 95% confidence interval, and declared the distributions different. If we model the distributions as normal distributions, the standard deviation increases from 4.65 dB with two MAVs to 4.9 dB with three MAVs. Aside from this, we also attribute the drop in relative localization performance (and the slower convergence) with three MAVs to the factors below.

- As also seen in the simulations from Sect. 6, flying with three MAVs pushes them further apart. This also happened in the real-world experiments, as can be seen in Fig. 18b. At larger distances, RSSI is less informative as a distance measurement due to its logarithmic decline.
- With three MAVs, the MAVs move slower and with more oscillations. This has a negative impact on the EKF’s performance, which favors smooth trajectories with faster velocities. A more aggressive MAV controller could have lowered this effect.
- The bearing error appears to diverge towards $\pm \,\pi$ more frequently with three MAVs. As already discussed in Sect. 3.5, this happens at smaller distances because a small error in *x* or *y* can translate into a large bearing error. When flying with three MAVs, the MAVs were also closer on more occasions than during flights with two MAVs. This can be seen in Fig. 18b.

## Experiments featuring on-board own-state measurements

The experiments from the controlled flights were repeated but with on-board state estimation by the MAVs. Therefore, on-board MAV sensors measured velocity, orientation, and height. This shows real-world relative localization performance for collision avoidance.

**Fig. 19:**
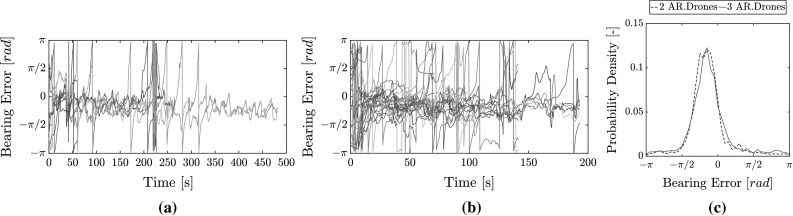
Overview of relative bearing estimation errors for flights with two AR.Drones and three AR.Drones featuring on-board own-state estimation. **a** With two AR.Drones, **b** with three AR.Drones, **c** bearing error distribution for all flights

### Experimental set-up

Velocity was estimated using the bottom facing camera and the *EdgeFlow* algorithm (McGuire et al. 2016). In the experiments described in this section, this provided velocity with a standard deviation between 0.10 and 0.35 m/s, as extracted by comparison with ground-truth measurements.^5^ The magnetometer could not be used due to large electro-magnetic disturbances in the environment, so orientation was measured using gyroscope integration only (given an initial orientation towards North). Height from the ground was measured using sonar. Optitrack was *only* used to enforce condition M1 (wall detection) with $d_{\mathrm {safe}}=0.5$ m. This is because wall detection is outside of the purpose of this research. To further stress-test the system, a further change was that the EKFs initial relative position assumption was $x_{ji}=y_{ji}=1$ m for any MAV ${\mathcal {R}}_i$ with respect to any other ${\mathcal {R}}_j$, as opposed to the center of the arena. The AR.Drones communicated with a ground-station using a Wi-Fi link for logging and take-off/land control.

### Results

Four flights were performed with two AR.Drones for a cumulative flight time of 17.3 min. In this time, only two collisions took place (after 3.9 and 1.0 min in the first and last flight). Another flight saw a near-collision in the early stages, but afterwards successfully continued until battery depletion. Another four flights were conducted with three AR.Drones, which lasted 8.3 min cumulatively. In this time, the MAVs experienced three collisions (after 1.2, 1.6, and 2.3 min of flight).

The bearing estimation error is shown in Fig. 19. The error has increased with comparison to the previous results. With two AR.Drones, the mean RMSE over the first three flights is 0.85 rad. This is sufficient for a long collision-free flight time. In the last flight, however, the RMSE was 1.3 rad, possibly due to a large disturbances in RSSI by the environment. This is held responsible for the early collision after 1.0 min. With three drones, the bearing RMSE over all flights is 1.0 rad. Figure 19c shows the distribution. There is an apparent negative bias. We can note how this bias increases in magnitude over time when observing Fig. 19a, b. This is due to the accumulating gyroscope bias during the flights. This could be corrected in future implementations by using a magnetometer.

## Implementing the technology on miniature drones

**Fig. 20:**
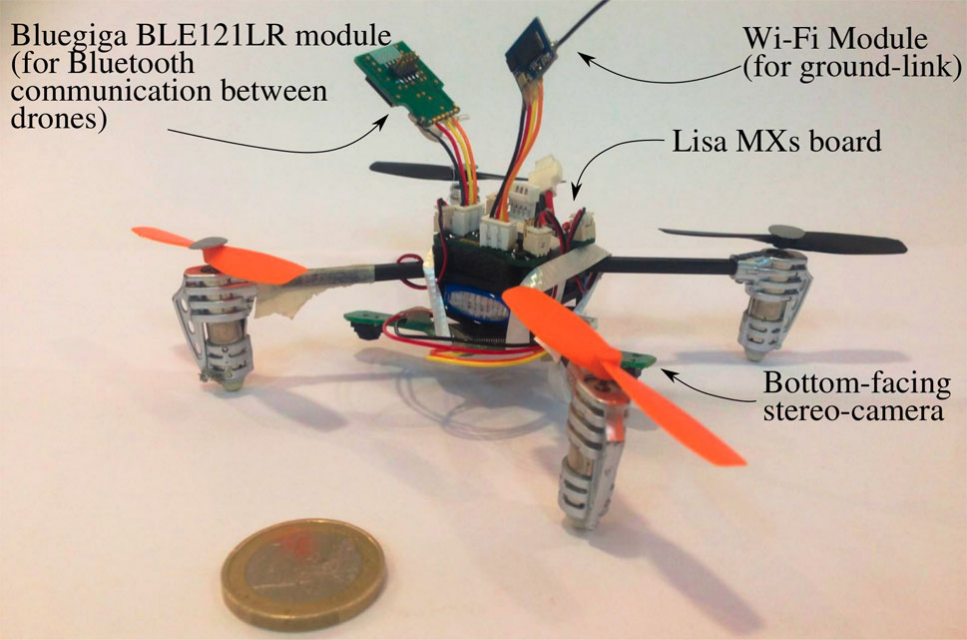
Miniature drone used in the experiments

To show that the proposed solution scales to smaller MAVs, we ported the technology to a pocket-sized MAV. A test-ready MAV and its components are shown in Fig. 20. The platform is as used by McGuire et al. (2017).

### Experimental set-up

The set-up is equivalent to the one for the AR.Drones in Sect. 8, with a few minor differences. In the LD model, following a short hand-held calibration, $P_n=-\,55$ dB. Also, given the smaller size of the drones, the enforced flight arena was reduced to $2~\mathrm{m}\times 2~\mathrm{m}$ with $d_{\mathrm {safe}}=0.5$ m. This scenario is similar to configuration 2 from Fig. 10. As for the AR.Drones in Sect. 8, a bottom facing camera and a gyroscope measured velocity and orientation, respectively. However, given the lack of an accurate height sensor on these small drones, the height was measured using Optitrack. Because of their fragility, the two drones also flew at different heights (1.0 and 1.5 m) so as to limit damages in case of failure.

### Results

Three flights were performed with this set up lasting 2.8, 3.7, and 3.1 min. The first flight saw no collision cases. The second flight saw near collisions at 1.35 and 3.7 min. The latter came in light of low-batteries by one of the drones. As it lowered its height, the two MAVs also actually collided. The third flight saw a near collisions after $\approx ~60$ s and $\approx ~90$ s. Both took place in the corner when condition M1 takes over the drones, and are thus regarded more as a failure of the behavior than the relative localization. This shows the importance of implementing a method that keeps taking into account other drones while also avoiding the walls, which was not implemented in our controller.

It is noted that a slightly lower performance than previous experiments was expected due to the smaller arena size, an effect which was also observed in simulation and is discussed further in Sect. 10.2. Nevertheless, we also note a decrease in accuracy for relative localization as RMSE per flight ranges from 0.8 to 1.37 rad. Inspecting the data in more detail shows that this is the result of larger errors in both RSSI noise (standard deviation 5.4–6.5 dB with mean error 2.1–4 dB) as well as lower quality of on-board velocity estimates (standard deviation of $\approx ~0.8$ m/s with mean error of up to 0.2 m/s). The former is explained by the fact that the Bluetooth module was placed right next to the Wi-Fi module, creating larger disturbances. The latter is explained by the lower resolution of the camera and the fact that it is subject to larger vibrations (McGuire et al. 2016). Furthermore, as the MAVs were flying closer to each other in a smaller arena, errors in relative *x* and *y* estimates are amplified when translated into relative bearing estimates.

## Discussion

### Performance of relative localization

In all AR.Drone tests, a loss in relative localization performance was measured when introducing a third MAV. The effects were longer convergence times as well as higher relative bearing/range errors. A decrease in performance was also observed when using on-board velocity estimates. This was due to a combination of over/under-estimation of velocity or occasional spikes in the measurements.

The relative localization scheme was implemented with an EKF. This may be criticized for its reliance on a Gaussian noise model. Robust (Kallapur et al. 2009) or adaptive (Sasiadek and Wang 1999) variants of Kalman filters, or particle filters (Svečko et al. 2015), might be better suited. However, a change in filter could increase computational costs without bringing a higher quality estimate. This is because there are a number of other limitations:

- The logarithmic decrease in RSSI makes it intrinsically insufficient to measure changes in range at larger distances.
- RSSI disturbances in the environment cannot be fully modeled unless the environment is known a-priori.
- The proposed process update equation makes the null assumption that all velocities remain constant between time-steps. Improvements may come from including more complex dynamic properties in the process equation, such as acceleration and/or jerk.
- As seen throughout our tests, improvements can come by improving the quality of on-board state estimates.

Further investigations are encouraged to define a filter that lowers the expected error.

We expect the main improvement to come from a change in communication hardware. In this work, we have achieved promising results using Bluetooth, which was selected due to its prompt availability on several MAVs. The noise and disturbances with Bluetooth ranging, however, are large. Other hardware, such as UWB, would offer a significant reduction in noise, leading to better overall relative localization results. Based on our simulations from Sect. 6.3, this should result in improved collision avoidance.

### Performance of collision avoidance

**Fig. 21:**
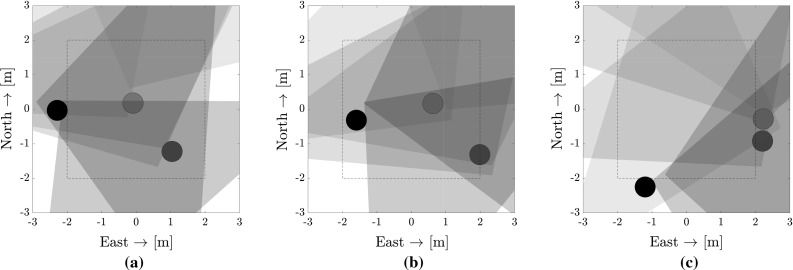
Chronological depiction (left to right) of a collision case in a flight with 3 AR.Drones (large circles indicate the ground-truth position in the arena, the triangles are the collision cones that each AR.Drone holds). **a** $\mathrm{Time }= 180\,\mathrm{s}$, **b** $\mathrm{time} = 182\,\mathrm{s}$, **c** $\mathrm{time} = 188\,\mathrm{s}$

The aim is to achieve a solution where no collisions will occur between the MAVs. In simulation, all twelve configurations have also been tested *without* active collision avoidance. The obtained mean flight times ranged between 3.9 and 14.3 s, as opposed to the results from Fig. 11. A *z*-test with $95\%$ confidence level (Dekking 2005) shows a statistically significant improvement in flight time for all configurations when using our method.

Figure 12a showed that smaller rooms lead to poorer performance than larger rooms despite similar airspace density. Reasons for this are:

- The ratio of arena size to $v_{\mathrm {nominal}}$ decreases in smaller rooms.
- The communication rate is constant, which limits the decision rate of the collision avoidance controller.
- In smaller rooms, M1 is called more frequently, in which case collision cones are ignored according to the task in this article.

It was observed that collisions for flights with three MAVs likely occur along the edges of the area. In the simulations of configuration 11, which is the one tested with the AR.Drones, $81\%$ of the collided simulated flights with three MAVs ended within 0.5 m of the arena borders. By comparison, only $35\%$ of collisions with two MAVs occurred within this space. This difference can also be appreciated visually in Fig. 13. An example of a collision extracted from an AR.Drone flight is recounted by the three events below (depicted in Fig. 21).

1. One MAV is at the corner and reluctant to make movements towards the center, gathering an oscillatory behavior. At time $t=180$ s (Fig. 21a), we see this for the bottom right AR.Drone (blue). Its slow speed causes the red MAV to mistaken its estimate of the blue drone. In normal conditions, collision avoidance could still be achieved by the blue MAV, but it cannot react as it is trapped in the corner.
2. Another MAV turns towards the same side. In time $t=182$ s (Fig. 21b), the central AR.Drone (red) avoids the black AR.Drone (on left) but in doing so goes to the right.
3. The second MAV also ends along the border and is reluctant to make movements. At time $t=188$ s (Fig. 21c), the two oscillate along the border until a collision occurs.

This scenario is less likely with two MAVs due to the larger freedom of movement and the generally higher relative localization accuracy. One method to limit this would be to reduce the angle of the collision cones for further away MAVs, increasing mobility. In the extreme, each MAV could only consider other MAVs within a close range. This could also help to increase the scalability of the method, so that only a small amount of parallel filters need to run even for large team sizes. Furthermore, it is also necessary to create an avoidance scheme that takes into account the wall and the drones together. This shall be tackled in future work.

## Conclusion and future work

We have shown that it is possible to use wireless communication as a relative localization sensor that can be used on-board of MAVs operating in a team. This leads to a large reduction in collisions without the need of a dedicated sensor. With the solution proposed in this paper, teams of two and three AR.Drones could fly in a $4~\mathrm{m}\times 4~\mathrm{m}$ area for minutes without collisions, despite being prompted to fly towards the center of the arena. This extreme test condition shows the high potential of our solution. The technology was also used with miniature drones, showing its portability. With respect to the scenario in mind (i.e. the exploration of indoor spaces by MAV teams), this is an efficient method to limit collision risks in the event that MAVs end up flying in the same room.

The combined relative localization/collision avoidance system as presented and tested in this paper will be further improved in future work. Importantly, we will investigate UWB modules instead of Bluetooth modules. Using UWB is expected to considerably improve the distance measurements used by the filter. The increase in accuracy could enable more complex group behaviors, such as formation flight, and accommodate larger teams of MAVs. This can extend the use of the system beyond the scope of this paper. Larger teams intrinsically occupy larger areas, and UWB can provide range measurements at larger distances without the degradation of Bluetooth RSSI. For true scalability to larger teams, there also needs to be a more detailed assessment of the communication algorithm. Our STDMA implementation has shown that it can work for small teams, but we cannot say whether it is the best solution as the size of the team grows. Furthermore, the introduction of an avoidance strategy that makes a more informed decision near walls or when multiple MAVs are present is needed. This could resolve the more complex collision scenarios, especially in smaller rooms. There is also on-going work to improve the miniaturized platforms to feature a front-facing camera, as in (McGuire et al. 2017), and active wall-sensors, so as to achieve fully autonomous teams of MAVs capable of exploring an unknown environment safely.

## Videos

Videos of experiments are available at: https://www.youtube.com/playlist?list=PL_KSX9GOn2P9f0qyWQNBMj7xpe1HARSpc.

## Footnotes

1 Experimentally, it has been found that office buildings can feature $2 \le \gamma _l \le 6$ (Kushki et al. 2008). Performing a sensitivity analysis of the LD model shows that an accurate identification of $\gamma _{l}$ has a low impact on the distance estimate at small distances.

2 An intuitive explanation of the observability is as follows: if robot ${\mathcal {R}}_i$ is moving towards North with 1 m/s and its distance to ${\mathcal {R}}_j$ (which, in this example, remains stationary) increases by 1 m each second, then ${\mathcal {R}}_j$ could infer that ${\mathcal {R}}_i$ is to its North. Similarly, ${\mathcal {R}}_i$ would know that ${\mathcal {R}}_j$ is to its South. This logic can be extended for all directions.

3 Note that, equivalently, the search could be anticlockwise, as long as this is equal for all agents.

4 Interestingly, emergent circular behaviors of this kind have also been observed in nature over a vast amount of organisms, ranging from bacteria to humans (Delcourt et al. 2016).

5 The differences arise from the fact that the noise is partially dependent on what the MAV sees during a flight, which depends on its chosen flight path.
